# The Impact of 5-Aminolevulinic Acid Supplementation on Redox Balance and Aerobic Capacity

**DOI:** 10.3390/ijms25020988

**Published:** 2024-01-12

**Authors:** Norio Saga, Ailing Hu, Takuji Yamaguchi, Yuna Naraoka, Hiroyuki Kobayashi

**Affiliations:** 1 Institute of Sports Science & Medicine, Teikyo University, Tokyo 173-8605, Japan; 2 Department of Personalised Kampo Medicine, Juntendo University Graduate School of Medicine, Tokyo 113-8421, Japan; ailing@juntendo.ac.jp (A.H.); tkyamagu@juntendo.ac.jp (T.Y.); 3 Intractable Disease Research Center, Juntendo University Graduate School of Medicine, Tokyo 113-8421, Japan; ynaraoka@juntendo.ac.jp; 4 Department of Hospital Administration, Juntendo University Graduate School of Medicine, Tokyo 113-8421, Japan; koba@juntendo.ac.jp

**Keywords:** 5-ALA, supplement, oxidative stress, antioxidant potential, aerobic exercise

## Abstract

We examined the impact of 5-aminolevulinic acid (5-ALA) and sodium-ferrous-citrate supplementation on aerobic capacity and redox balance through a placebo-controlled, double-blind trial. Fourteen healthy volunteers were randomly assigned to Pla + ALA (4-week placebo followed by 4-week 5-ALA supplementation) or ALA + Pla (4-week 5-ALA supplement followed by a 4-week placebo) group and administered 5-ALA (25 mg/day) or placebo once daily. The participants underwent submaximal incremental cycling tests at weeks 0, 2, 4, 6, and 8. In the cycling test at week 0, individual load-intensity stages required for blood lactate levels >2 mmol/L (lactate threshold, LT) and 4 mmol/L (onset of blood lactate accumulation, OBLA) were determined. The heart rate (HR), blood lactate (La), and oxidative stress markers (diacron reactive oxygen metabolite, d-ROMs; biological antioxidant potential, BAP) were measured at resting, LT, and OBLA states in each cycling test. Marker values were not significantly different between the groups. HR, La, and d-ROMs at resting, LT, and OBLA states were not significantly different among the conditions. BAP and BAP/d-ROMs ratios were significantly different in the OBLA state at week 4 of the 5-ALA group compared with that of the placebo group (*p* < 0.05). In conclusion, 5-ALA supplementation might improve redox balance during high-intensity aerobic exercise.

## 1. Introduction

5-aminolevulinic acid (5-ALA), generated in mitochondria, is the first product of the porphyrin synthesis pathway. Furthermore, 5-ALA is a precursor of heme, which plays an important role in aerobic energy metabolism. However, the amount of 5-ALA produced gradually decreases with age. 5-ALA is a naturally occurring amino acid present in very small quantities in various foods, including spinach (0.18 mg/kg), green peppers (0.23 mg/kg), tomatoes (0.13 mg/kg), bananas (0.4 mg/kg), octopus (1.0 mg/kg), and ground beef (0.13 mg/kg) [1].

5-ALA supplementation has been shown to improve the activity of the mitochondrial electron transport system and the production of adenosine triphosphate in mouse livers [2]. Rodriguez et al. [1] reported that 5-ALA reduced fasting blood glucose levels in prediabetic patients aged 40–70 years and improved glucose metabolism during a glucose tolerance test. Sato et al. reported that 5-ALA improved lipid metabolism and caused visceral fat accumulation in rats that were fed a high-fat diet [3]. Thus, 5-ALA supplementation is expected to help prevent the development of lifestyle diseases caused by metabolic and endocrine abnormalities.

Combination treatment of 5-ALA and sodium ferrous citrate (SFC) has been shown to enhance exercise efficiency and voluntary interval walking training in older women [4]. These findings suggest that 5-ALA dietary supplements improve mitochondrial function and exercise efficiency. However, it remains unclear whether daily 5-ALA supplementation without aerobic exercise training can increase aerobic capacity and protect against exercise-induced oxidative stress.

Exercise, even in single bouts, can increase the generation of reactive oxygen species (ROS) within human skeletal muscle, potentially diminishing the production of maximal force [5]. Oxidative stress levels and cytokine concentrations tend to exhibit moderate to severe increases following acute exercise [6]. Research has shown that prolonged exercise, such as triathlon competitions, may induce neutrophil apoptosis [7]. In addition, sustained training over a 4-week period has been suggested to induce oxidative stress, which may serve as an inadequate recovery marker leading to overreaching [8]. Furthermore, in overtraining, biomarkers of oxidative stress have shown responsiveness, with some makers proportional to training load, suggesting that oxidative stress may be a diagnostic tool for overtraining [9]. Increased oxidative stress is implicated in the pathophysiology of overtraining syndrome, and the reduced response of oxidative stress markers and antioxidant capacity to exercise during overtraining may be related to impaired adaptation to exercise [10]. Consequently, it might be important for athletes to monitor their oxidative stress levels closely.

In this study, a submaximal incremental cycling test was performed to examine the impact of daily 5-ALA (25 mg/day) and SFC (29 mg/day) supplementation on aerobic capacity and redox balance in young and healthy Japanese volunteers.

## 2. Results

### 2.1. Primary Analysis

In the primary analysis, we evaluated the impact of 5-ALA supplementation by comparing changes in objective and subjective parameters of both study groups over 8 weeks. Two-way repeated measures analysis of variance (ANOVA) revealed no significant differences between factors of group, time, or their interaction with any of the parameters.

### 2.2. Secondary Analysis

**Figure 1:**
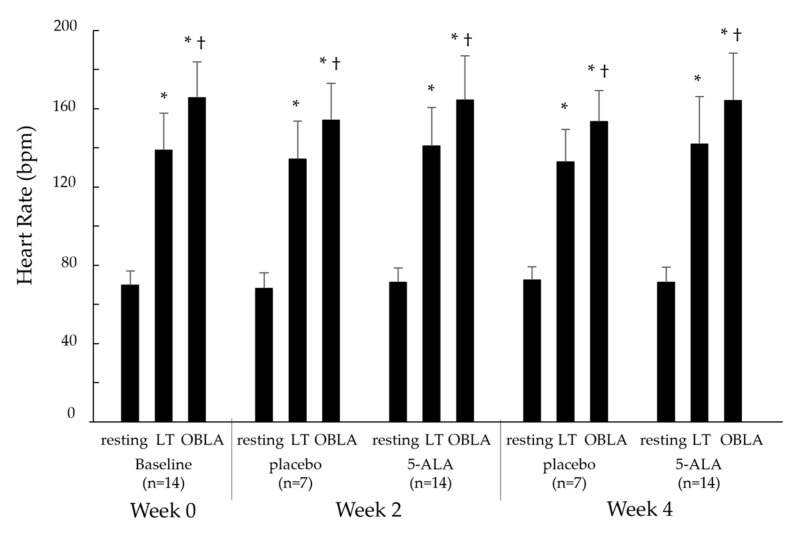
Changes in HR at resting, LT, and OBLA states in baseline, placebo, and 5-ALA conditions. Baseline condition: week 0; placebo condition: weeks 2 and 4; 5-ALA condition: weeks 2 and 4. HR: heart rate; LT: lactate threshold; OBLA: onset of blood lactate accumulation; 5-ALA: 5-aminolevulinic acid; * *p* < 0.05 vs. resting state, † *p* < 0.05 vs. LT state.

**Figure 2:**
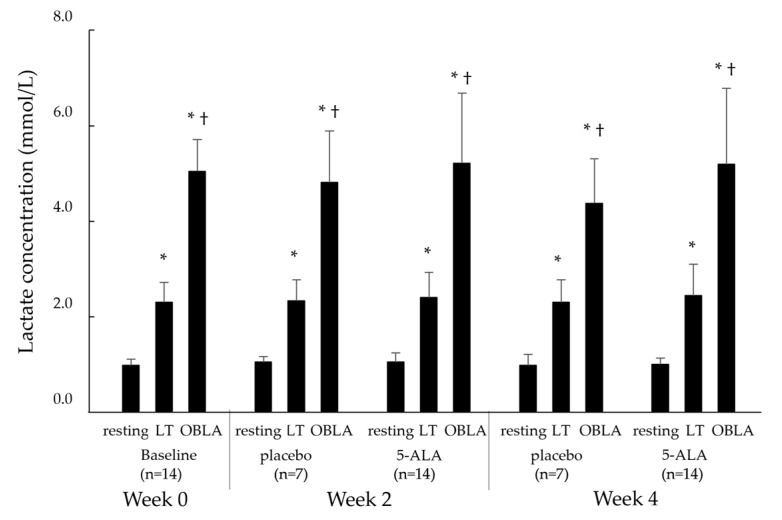
Changes in blood lactate concentrations at resting, LT, OBLA states in baseline, placebo, and 5-ALA conditions. Baseline condition: week 0; placebo condition: weeks 2 and 4; 5-ALA condition: weeks 2 and 4. LT: lactate threshold; OBLA: onset of blood lactate accumulation; 5-ALA: 5-aminolevulinic acid; * *p* < 0.05 vs. resting state, † *p* < 0.05 vs. LT state.

**Figure 3:**
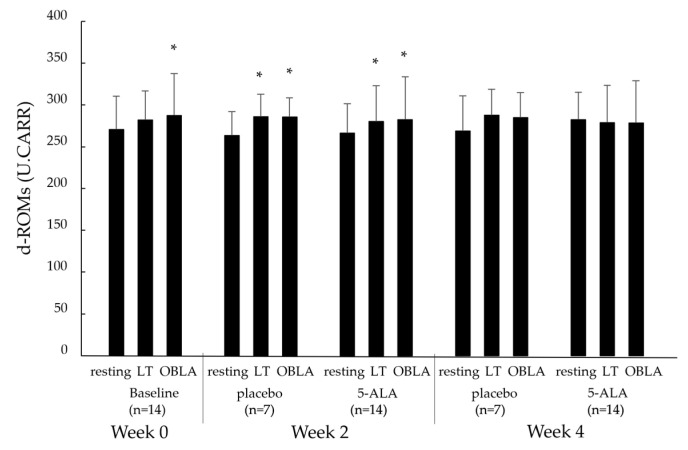
Changes in blood d-ROM levels at resting, LT, OBLA states in baseline, placebo, and 5-ALA conditions. Baseline condition: week 0; placebo condition: weeks 2 and 4; 5-ALA condition: weeks 2 and 4. d-ROMs: diacron reactive oxygen metabolite; LT: lactate threshold; OBLA: onset of blood lactate accumulation; 5-ALA: 5-aminolevulinic acid; * *p* < 0.05 vs. resting state.

**Figure 4:**
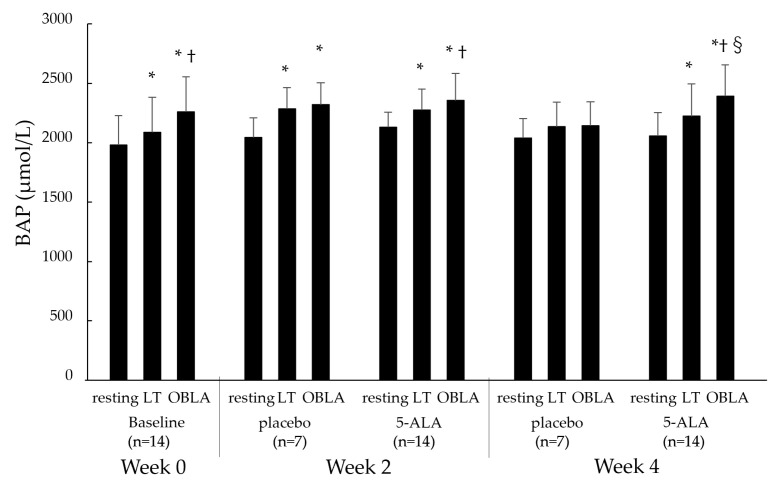
Changes in BAP levels at resting, LT, OBLA states in baseline, placebo, and 5-ALA conditions. Baseline condition: week 0; placebo condition: weeks 2 and 4; 5-ALA condition: weeks 2 and 4. BAP: biological antioxidant potential; LT: lactate threshold; OBLA: onset of blood lactate accumulation; * *p* < 0.05 vs. resting state, † *p* < 0.05 vs. LT state, § *p* < 0.05 vs. placebo.

**Figure 5:**
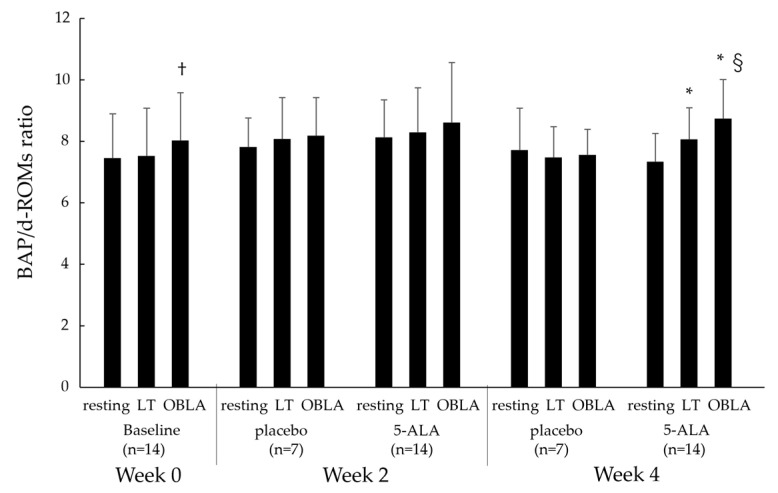
Changes in BAP/d-ROMs ratios at resting, LT, OBLA states in the baseline, placebo, and 5-ALA conditions. Baseline condition: week 0; placebo condition: weeks 2 and 4; 5-ALA condition: weeks 2 and 4. BAP: biological antioxidant potential; d-ROMs: diacron reactive oxygen metabolite; LT: lactate threshold; OBLA: onset of blood lactate accumulation; * *p* < 0.05 vs. resting state, † *p* < 0.05 vs. LT state, § *p* < 0.05 vs. placebo.

In the secondary analysis, we evaluated the impact of the 4-week 5-ALA supplementation, in which data from both 5-ALA conditions were integrated (n = 14) and compared with baseline (n = 14) and placebo data (n = 7). The results of the secondary analysis for all objective and subjective parameters at the resting lactate threshold (LT) and the onset of blood lactate accumulation (OBLA) states in the baseline, placebo, and 5-ALA conditions are shown in Figure 1, Figure 2, Figure 3, Figure 4 and Figure 5.

#### 2.2.1. Heart Rate

Figure 1 shows the heart rate (HR) during the cycling test at the resting, LT, and OBLA states in the baseline (week 0), placebo (weeks 2 and 4), and 5-ALA (weeks 2 and 4) conditions. HR in all conditions showed a significant increase, with an increase in pedalling load, from the resting to LT states (*p* < 0.05), resting to OBLA states (*p* < 0.05), and LT to OBLA states (*p* < 0.05). However, HRs in the 5-ALA condition at weeks 2 and 4 were not significantly different from those in the baseline and placebo conditions.

#### 2.2.2. Blood Lactate Concentrations (La)

Figure 2 shows the La during the cycling test at the resting, LT, and OBLA states in the baseline (week 0), placebo (weeks 2 and 4), and 5-ALA conditions (weeks 2 and 4). La in all conditions showed a significant increase, with an increase in pedalling load, from the resting to LT states (*p* < 0.05), resting to OBLA states (*p* < 0.05), and LT to OBLA states (*p* < 0.05). However, La levels in the 5-ALA condition at weeks 2 and 4 were not significantly different from those in the baseline and placebo conditions.

#### 2.2.3. Diacron Reactive Oxygen Metabolites (d-ROMs) and Biological Antioxidant Potential (BAP)

Figure 3 shows the blood d-ROM levels during the cycling test at the resting, LT, and OBLA states in the baseline (week 0), placebo (weeks 2 and 4), and 5-ALA (weeks 2 and 4) conditions. The d-ROM levels in the baseline condition showed a significant increase, with an increase in pedalling load, from the resting to OBLA states (*p* < 0.05). The d-ROM levels in the placebo and 5-ALA conditions at week 2 showed a significant increase, with an increase in pedalling load, from the resting to LT and OBLA states (*p* < 0.05). However, no significant differences were observed in d-ROM levels in the placebo or 5-ALA conditions at week 4. Changes in d-ROM levels in the 5-ALA condition at weeks 2 and 4 were not significantly different from those in the baseline and placebo conditions.

Figure 4 shows the blood BAP levels during the cycling test at the resting, LT, and OBLA states in the baseline (week 0), placebo (week 2), and 5-ALA (weeks 2 and 4) conditions. The BAP levels in all conditions except for the placebo condition at weeks 2 and 4 showed a significant increase, with an increase in pedalling load, from the resting to LT states (*p* < 0.05) and LT to OBLA states (*p* < 0.05). The BAP levels in the 5-ALA condition increased at 0 and 2 weeks compared with the placebo condition, but there was no significant change (resting: *p* = 0.09, LT: *p* = 0.06). However, the BAP level in the OBLA state of the 5-ALA condition at week 4 was significantly increased compared with that of the placebo condition at week 4 (*p* < 0.05).

Figure 5 shows the BAP/d-ROMs ratios during the cycling test at the resting, LT, and OBLA states in the baseline (week 0), placebo (weeks 2 and 4), and 5-ALA (weeks 2 and 4) conditions. The BAP/d-ROMs ratio in the 5-ALA condition at week 4 showed a significant increase, with an increase in pedalling load, from the resting to LT and OBLA state (*p* < 0.05). The BAP/d-ROMs ratios at 2 weeks in the 5-ALA condition were higher than the placebo condition at 0 weeks, but there was no significant change (resting: *p* = 0.08, LT: *p* = 0.06). In addition, the OBLA state of the BAP/d-ROMs ratios at 4 weeks in the 5-ALA condition was higher than the placebo condition at 0 weeks, but there was no significant change (*p* = 0.08). However, the BAP/d-ROMs ratio in the OBLA state of the 5-ALA condition at week 4 was significantly increased compared with that of the placebo condition at week 4 (*p* < 0.05).

#### 2.2.4. Salivary Cortisol and Secretory Immunoglobulin A Levels (s-IgA)

Salivary cortisol levels at the resting, LT, and OBLA states in the 5-ALA condition at week 4 were 0.15 ± 0.14, 0.13 ± 0.12, and 0.11 ± 0.09 μg/dL, respectively. These cortisol levels showed no significant differences compared with those of the placebo condition at week 4 (0.15 ± 0.17, 0.11 ± 0.11, and 0.10 ± 0.10 μg/dL, respectively).

Salivary s-IgA levels at the resting, LT, and OBLA states in the 5-ALA condition at week 4 were 41.8 ± 29.4, 93.7 ± 62.1, and 93.9 ± 39.9 μg/mL, respectively. At 4 weeks in the 5-ALA condition, the salivary s-IgA levels showed lower values than the placebo condition, but there was no significant change (*p* = 0.08). These s-IgA levels were not significantly different from those of the placebo condition at week 4 (72.4 ± 50.4, 78.1 ± 46.5, and 135.9 ± 64.3 μg/mL, respectively).

#### 2.2.5. Rating of Perceived Exertion (RPE)

The RPE in all conditions showed a significant increase, with an increase in pedalling load (*p* < 0.05). The RPE showed lower values at 2 weeks compared to 0 weeks in the 5-ALA condition, but there was no significant change (*p* = 0.07). However, the RPE in the 5-ALA condition at weeks 2 and 4 was not significantly different in the LT or OBLA states in the placebo conditions.

#### 2.2.6. Profile of Mood States

Table 1 shows the profile of mood states in the baseline (week 0), placebo (weeks 2 and 4), and 5-ALA (weeks 2 and 4) conditions. No significant differences in any of the seven parameters were observed in the 5-ALA conditions (weeks 2 and 4) compared with those in the placebo (weeks 2 and 4) and baseline (week 0) conditions.

#### 2.2.7. Fatigue

The visual analog scale (VAS) score in the baseline condition (week 0) was 39 ± 20 mm. The VAS scores in the placebo condition at weeks 2 and 4 were 44 ± 11 and 46 ± 21 mm, respectively. The VAS scores in the 5-ALA condition at weeks 2 and 4 were 43 ± 21 and 41 ± 18 mm, respectively. No significant differences were observed between the VAS scores in the 5-ALA conditions (weeks 2 and 4) and those of the placebo (weeks 2 and 4) and baseline (week 0) conditions.

## 3. Discussion

This study examined the impact of 5-ALA supplementation on the aerobic capacity, oxidative stress, and antioxidant potential. The primary analysis failed to document the impact of 5-ALA supplementation on the objective and subjective parameters in either of the groups over 8 weeks. However, a secondary analysis revealed that the BAP and BAP/d-ROMs ratios increased significantly after 4 weeks of 5-ALA supplementation. Nevertheless, the d-ROMs did not decrease after 4 weeks of ALA supplementation. These findings suggest that daily 5-ALA intake, regardless of its combination with exercise, may improve redox balance during high-intensity aerobic exercise in young adults.

### 3.1. Oxidative Stress and Antioxidant Potential

5-ALA is an intermediate in the heme synthesis pathway. Heme is used to produce haemoglobin and cytochromes, which are essential for mitochondrial energy production [11]. 5-ALA induces the upregulation of heme oxygenase-1 (HO-1) mRNA [12,13]. HO-1 expression is induced by various stressors and degrades heme into biliverdin, carbon monoxide (CO), and iron. HO-1 has antioxidative and anti-inflammatory functions via the actions of biliverdin and CO, respectively [14,15]. In addition, 5-ALA reduces fasting and postprandial glucose levels in mildly hyperglycaemic participants, and oral 5-ALA administration may also be a novel approach for the prevention of type 2 diabetes [16]. Hou et al. reported that the protective effects of pretreatment with 5-ALA combined with ferrous iron were associated with its antioxidant, anti-inflammatory, and anti-programmed-cell-death mechanisms in mice [12]. Thus, 5-ALA intake may reduce oxidative stress during relatively high-intensity exercise, which was reflected in our study. In this study, the d-ROMs did not change after 4 weeks of ALA supplementation. In this regard, the BAP and BAP/d-ROMs ratios, which reflect the antioxidant capacity, were significantly increased in exercise-induced OBLA states in participants who received 5-ALA treatment for 4 weeks.

Moreover, modest elevations in skeletal muscle ROS levels, such as during mild to moderate exercise, have been suggested to improve, or even be required for, contractile function [17]. Therefore, it may be better to combine moderate exercise throughout the dosing period to understand the impact of 5-ALA supplementation on redox balance improvement precisely. In this study, while the participants did not receive a combination of exercises during the 5-ALA dosing period, the results suggested that high-intensity aerobic exercise improved antioxidant potential and redox balance.

Exercise, particularly in single bouts, can increase ROS generation, which indicates oxidative damage of DNA (8-hydroxydeoxyguanosine), thereby decreasing maximal force generation [5]. Varamenti et al. suggested that the changes in oxidative stress and cytokine levels after acute exercise ranged from moderate to extremely large and that the variation after chronic exercise ranged from trivial to moderate [6]. Tryfidou et al. reported that DNA damage increased after high-intensity exercise (≥75% VO_2_max) [18]. Lewis et al. suggested that an overwhelming increase in ROS generation led to increased cell apoptosis and immunosuppression, which in turn resulted in underperformance [19].

High levels of ROS production could damage proteins, lipids, and DNA. However, Tsuzuki et al. reported that the redox balance shifted antioxidation during a duathlon and that the increased oxidant potential levels correlated negatively with performance in the early stages of the race [20]. Also, moderate-exercise-induced ROS could promote mitochondrial biosynthesis as well as the synthesis of antioxidant enzymes and stress proteins in skeletal muscle [21]. In the present study, 5-ALA did not inhibit oxidative stress but increased it on par with a placebo and increased the antioxidant capacity. In other words, while oxidative stress, which is necessary for exercise adaptation, is maintained, the increased antioxidant capacity leads to non-interference with the after-effects of acute exercise, which is an important result in this study.

Our results suggest that 5-ALA supplementation might improve redox balance during high-intensity aerobic exercise, which may prevent performance deterioration and improve subsequent recovery. Therefore, daily 5-ALA supplementation may help with sports preconditioning. Further research is necessary to confirm these findings.

### 3.2. Aerobic Capacity

Masuki et al. demonstrated that 5-ALA (with SFC) supplements increased the exercise efficiency and voluntary interval walking training in older women. They suggested that ALA supplementation improved mitochondrial function (via restoring the age-associated decrease in transient oxygen utilisation rates) and exercise efficiency, measured as work per total metabolic cost of exercise [4]. They also reported that an increase in lactic acid was attenuated significantly (by 16%) at every workload stage in their 5-ALA trial in older participants [4]. In contrast, our study demonstrated no impact of 5-ALA supplementation on lactate levels. We speculate that this discrepancy might be related to the relatively younger age (university students) of our study participants. Oxygen consumption also decreases with age because muscle mitochondria and/or their function are reduced with age [22,23]. Conversely, oxidative stress increases with age [24]. As oral 5-ALA supplementation has been reported to improve exercise efficiency in older women [4], 5-ALA supplementation may be more effective in activating mitochondrial function in older people. In contrast, younger participants are considered to have a stronger defence system against exercise-induced oxidative stress than older adults. Additionally, erythrocytes have potent antioxidant protection consisting of enzymatic and non-enzymatic pathways that counteract reactive oxygen species, thus maintaining redox regulation in the body. However, during human aging, erythrocyte membrane-SH groups, which play a major role in maintaining the oxidation-reduction status of the cell, decrease [25]. Therefore, in this trial on young participants, the impact of 5-ALA supplementation on HR, La, and d-ROMs may have been less pronounced compared to the placebo impact. However, BAP and BAP/d-ROM levels were significantly increased by 5-ALA treatment, suggesting that 5-ALA activates mitochondria and increases antioxidant capacity, thereby improving the redox balance. In addition, the results of this study might have been affected by sex hormones because of the inclusion of female participants in this study. Oestrogen may have protective effects on cardiac, smooth, and possibly skeletal muscle in terms of oxidative damage and inflammation. It also has a high antioxidant capacity [25]. Therefore, the impact of 5-ALA supplementation may have been potentially masked by that of oestrogen on the antioxidant capacity in females. In addition, the 5-ALA dosage might have influenced the results in female participants. In other words, 25 mg of 5-ALA was used in this study, whereas the previous study involving older women used a dose of 50 mg of 5-ALA [4], which has been reported to be safe and effective for participants engaging in aerobic exercise [4,26]. Therefore, future studies should examine the relationships among age, sex, and 5-ALA dosage. Investigating whether 5-ALA increases endurance and improves the redox balance when combined with exercise, even in young men, could be valuable for athletes of all ages.

### 3.3. Profile of Mood States and Fatigue

The present study also examined whether daily 5-ALA supplementation caused fatigue and changes in the short Japanese version of the Profile of Mood States, 2nd edition (POMS 2-Brief form) score. Aquino et al. investigated the impact of 5-ALA supplementation on mood and coping ability in prediabetic middle-aged and older adults using the Psychosocial Depressive Symptoms Questionnaire and Perceived Stress Scale. Their findings suggested that 12-week treatment with 5-ALA improved the self-perception of effort spent, loneliness, and coping ability [27]. Lewis et al. suggested that an overwhelming increase in oxidative stress due to exercise load leads to an increase in fatigue [19]. Therefore, we examined the impact of 5-ALA supplementation on mood and fatigue in young and healthy volunteers. However, no significant effects were observed in our study. Therefore, future studies should assess the impact of 5-ALA supplementation on mood and fatigue in symptomatic participants.

## 4. Materials and Methods

### 4.1. Participants

**Figure 6:**
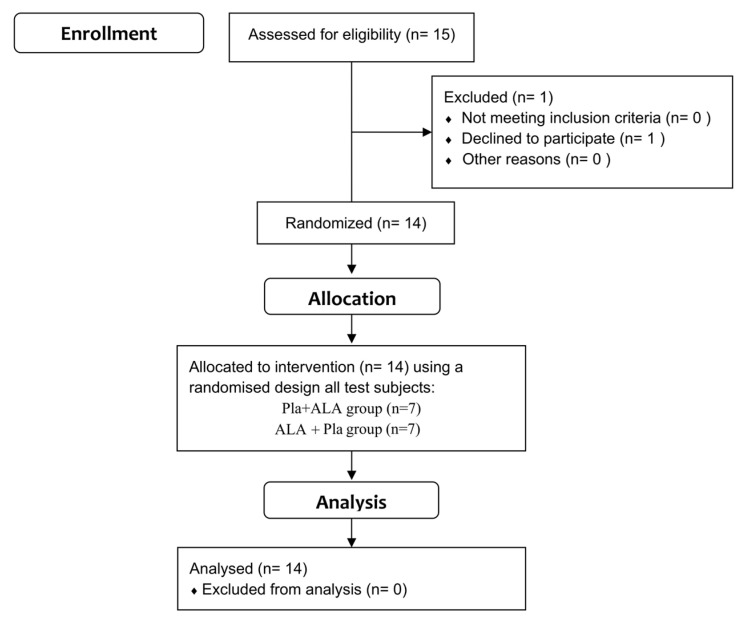
Flow diagram of the progress through the phases of a parallel randomised trial of the two groups. ALA + Pla group, 4-week 5-ALA supplement followed by a 4-week placebo; Pla + ALA group, 4-week placebo followed by a 4-week 5-ALA supplement.

This study recruited participants aged 20–40, excluding those using any type of medication, who had sedentary or recreational activity levels, were non-smokers, and were in good health. Fifteen participants were initially recruited and informed about the study, but one declined to participate. Subsequently, fourteen healthy volunteers, comprising seven men (mean age: 33 ± 11 years, range: 20–40 years; mean height: 173.9 ± 5.9 cm; mean weight: 70.9 ± 10.7 kg) and seven women (mean age: 21 ± 1 years; mean height: 161.0 ± 8.9 cm; mean weight: 56.0 ± 7.0 kg), voluntarily enrolled in this placebo-controlled, double-blind trial (Figure 6).

Although 5-ALA can be found in the daily diet, it is present in trace amounts. Therefore, no special dietary management regarding 5-ALA was conducted during the study period. Participants were also instructed to refrain from engaging in additional forms of exercise, taking dietary supplements, or undergoing medicinal interventions throughout the experimental period.

All participants provided informed consent for their participation in the study. This study was approved by the Juntendo University Human Ethics Committee (No. 18-005) and adhered to the principles of the Declaration of Helsinki.

### 4.2. Clinical Trial Design

**Figure 7:**
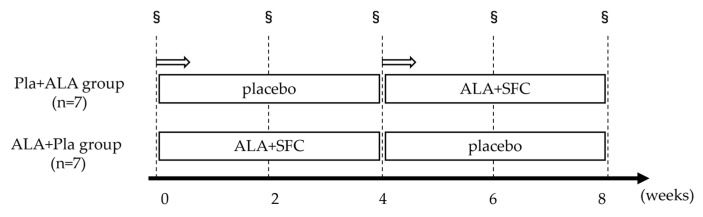
Experimental design. §: submaximal incremental cycling test.

The experimental design is illustrated in Figure 7. Participants (n = 14) were randomly assigned to either the Pla + ALA group (4-week placebo followed by 4-week 5-ALA supplementation) or the ALA + Pla group (4-week 5-ALA supplement followed by a 4-week placebo). The 5-ALA supplement (5-ALA 25 mg/day + SFC 29 mg/day) or the placebo (pregelatinised starch 230 mg/day + SFC 29 mg/day), which were supplied by SBI ALApromo (Tokyo, Japan), was taken once daily at night. Participants in each group underwent submaximal incremental cycling tests at weeks 0, 2, 4, 6, and 8. Furthermore, to account for the influence of diurnal variability on certain parameters, we instructed participants to visit the laboratory at the same time of day for each test. During the experimental period, the participants were prohibited from performing other forms of exercise, taking dietary supplements, or undergoing medicinal interventions.

Aquino R et al. reported that 5-ALA, administered in dosages of both 15 and 50 mg in combination with SFC, demonstrated safety and suitability as a dietary supplement for individuals with prediabetes [27]. Additionally, a previous study [28] showed that an intake of 30 mg of 5-ALA for 8 weeks resulted in improved feelings of chronic physical fatigue and a reduction in negative mood among men and women aged 20–64 who experienced daily physical fatigue and a negative mood profile. Therefore, we decided to use a moderate 25 mg of ALA, as used in the previous study.

SFC, as a source of iron ions, plays a crucial role in facilitating the final step of heme biosynthesis by interacting with the mitochondrial ABCB6 transporter and ferrochelatase. The inclusion of SFC in the supplement was designed to prevent the accumulation of heme biosynthesis intermediates, such as protoporphyrin IX, which might cause photodamage to the skin during outdoor exercise [4,29].

Furthermore, in a previous study conducted with a randomised, placebo-controlled, double-blind crossover design, there was no difference in the pre-values of both supplements in a 2-week washout study of 5-ALA and placebo supplementation for 1 week. In this study, there was no established washout period or specific dietary controls implemented for 5-ALA before the commencement of the study, as 5-ALA is found in trace amounts in the daily diet.

### 4.3. Submaximal Incremental Cycling Test

Submaximal incremental cycling tests at weeks 0, 2, 4, 6, and 8 were performed using a cycle ergometer (Ergomedic 894E; Monark, Vansbro, Sweden) at room temperature and humidity of 23.2 ± 1.2 °C and 52.1 ± 9.0%, respectively. The cycling test was programmed to increase the pedalling load by 0.5 kp every 3 min. In cycling tests performed on all participants prior to supplement intervention at week 0, the individual load intensity stage required blood lactate levels of >2 mmol/L (LT) and 4 mmol/L (OBLA). Thereafter, HR, blood lactate, d-ROMs, BAP, salivary cortisol, and s-IgA in each participant were measured at individual resting, LT, and OBLA states in each cycling test. All participants were instructed to fast for 3 h before their cycling test but were allowed to drink water freely.

Subjective assessments of mood profile and fatigue were recorded prior to each cycling test.

### 4.4. Measurements

#### 4.4.1. HR

HR was measured using an HR monitor (PolarM460; Polar Electro, Kempele, Finland) that was attached to the chest with a strap sensor. The average HR at the resting, LT, and OBLA states was determined by monitoring the last 15 s of the 3-min load exercise in each stage.

#### 4.4.2. La

Blood samples collected at 3-min intervals in the first incremental cycling test were used to determine LT and OBLA from La. Blood samples collected at the resting, LT, and OBLA states in subsequent cycling tests were measured using a blood lactate analyser (Lactate Pro 2, LT-1730; ARKRAY, Kyoto, Japan). This analyser operates on an enzymatic amperometric detection method [30]. It interprets the electrical signal produced due to the reaction between lactate in the blood and the enzyme lactate oxidase on the inserted sensor. The voltage signal directly corresponds to the lactate concentration of the samples, allowing measurement of blood lactate concentration.

Blood samples (approximately 0.3 μL) were collected from each participant’s fingertip after each incremental cycling load exercise for 3 min.

#### 4.4.3. d-ROMs and BAP

Approximately 200 μL of blood was collected from the fingertip of each participant and used for the analysis of d-ROMs and BAP, which are markers of oxidative stress [31,32]. The levels of d-ROMs and BAP were measured using a free radical analyser (FREE Carrio Duo; Wismerll, Tokyo, Japan) at the resting, LT, and OBLA states. The BAP/d-ROMs ratio was calculated to assess the redox balance.

In the d-ROMs test [32], reactive oxygen metabolites (primarily hydroperoxides) of a biological sample, in the presence of iron released from plasma proteins by an acidic buffer, can generate alkoxyl and peroxyl radicals via Fenton’s reaction. These radicals subsequently oxidise N,N-dietylparaphenylendiamine, producing a pink-coloured derivative quantified at 505 nm using spectrophotometry. The d-ROMs can be detected spectrophotometrically using an automatic analyser. The results of the d-ROMs analysis were expressed in Carratelli units (U. CARR.), where 1 U. CARR. is equivalent to 0.08 mg hydrogen peroxide/dL, primarily due to hydroperoxides, with the contribution of other minor oxidant factors.

In the BAP test [32,33] the addition of a plasma sample to a coloured solution, obtained by mixing a ferric chloride solution with a thiocyanate derivative solution, leads to decolouration. The intensity of this decolouration is measured photometrically at 505 nm and is proportional to the plasma’s ability to reduce ferric ions. The results are expressed as μmol/L and represent the biological antioxidant potential.

#### 4.4.4. Salivary Cortisol and s-IgA

Participants used saliva collection aids (SCA; Salimetrics, State College, PA, USA) to collect their saliva into collection vials. Salivary samples were stored at 20 °C until analysis by immunoassay kits (Salimetrics) for salivary cortisol and s-IgA levels. Salivary cortisol and s-IgA levels were determined using an enzyme-linked immunosorbent assay with the following kits, respectively: salivary cortisol enzyme immunoassay kit and secretory immunoglobulin A salivary immunoassay kit (Salimetrics LLC, Carlsbad, CA, USA) [34].

#### 4.4.5. RPE

The RPE of all participants was evaluated using the Borg scale, which ranges from 6 to 20 points [35].

#### 4.4.6. Profile of Mood States

The profile of mood states in all participants was evaluated using a POMS 2-Brief Form [36,37]. POMS2 is a tool for assessing distinct mood states. The full version of POMS2 consists of 65 questions and T-scores of 6 mood clusters. This version, consisting of 30 questions, assesses the following seven parameters: anger/hostility, confusion/bewilderment, depression/dejection, fatigue/inertia, tension/anxiety, vigor/activity, friendliness, and total mood disturbance. The POMS test is known to be used in the study of the relationship between mood and exercise or physical activity [37].

#### 4.4.7. Fatigue

Fatigue in all participants was assessed using a 100 mm VAS [38]. The left point (0 mm) on the VAS line indicates “no fatigue at all”, and the right point (100 mm) indicates being “extremely tired”. Patients marked their degree of malaise on the line, and the intensity of perceived fatigue was quantified by measuring the distance from 0 mm.

### 4.5. Statistical Analysis

#### 4.5.1. Primary Analysis

In the primary analysis, the impact of 5-ALA was analysed by comparing the changes in each parameter between both groups over 8 weeks. The statistical significance of the group, time, and their interaction (group × time) factors was assessed using two-way repeated measures ANOVA. The significance level (*p*) was set at *p* < 0.05. The sample size needed for the two-way ANOVA (alpha error = 0.05, power = 0.80, effect size = 0.25) was calculated, and it was determined that 22 participants would be required for this study.

#### 4.5.2. Secondary Analysis

In the secondary analysis, the data were divided into three parts: baseline, placebo, and 5-ALA. As in the previous report [39], the number of samples was increased by 200 (approximately 10%) in the BAP, with a standard deviation of 250. Subsequently, the sample size was determined to be 15. The baseline condition used data from all participants on day 0 (n = 14). The placebo condition used data (n = 7) from participants who received a 4-week placebo treatment in the Pla + ALA group. The 5-ALA condition used data from the primary analysis of all participants (n = 14). The impact of 5-ALA supplementation on each parameter was compared with those of the baseline and placebo conditions at the resting, LT, and OBLA states. The statistical significance of each parameter was assessed using a paired *t*-test. The significance level (*p*) was set at <0.05. Data are expressed as mean ± standard deviation.

## 5. Conclusions

This study examined the impact of 4-week 5-ALA supplementation on aerobic capacity, oxidative stress, and antioxidant potential. Although 5-ALA + SFC supplementation did not affect aerobic capacity, it might improve redox balance during high-intensity aerobic exercise in young people. Further research is required on increased 5-ALA intake and the potential synergy between 5-ALA and exercise to clarify the impact of 5-ALA on sports in young individuals.

## Figures and Tables

**Table 1:** Profile of mood states.

		Week 0	Week 2		Week 4	
	POMS 2 Scales	Baseline	Pla	ALA	Pla	ALA
Profile of Mood States	Anger/Hostility	3.1 ± 2.9	3.0 ± 2.4	4.9 ± 4.6	3.4 ± 2.3	4.2 ± 4.9
	Confusion/Bewilderment	7.3 ± 3.2	7.0 ± 3.8	6.1 ± 2.7	9.1 ± 4.4	6.6 ± 4.2
	Depression/Dejection	4.1 ± 3.1	4.3 ± 4.9	3.5 ± 2.8	4.9 ± 3.3	3.2 ± 3.6
	Fatigue/Inertia	6.4 ± 3.3	6.9 ± 2.8	6.9 ± 3.9	9.6 ± 3.8	7.1 ± 4.4
	Tension/Anxiety	6.9 ± 3.5	7.4 ± 4.3	6.8 ± 2.8	8.1 ± 4.9	5.9 ± 3.8
	Vigor/Activity	11.3 ± 4.5	12.0 ± 3.1	10.0 ± 4.0	9.0 ± 2.8	10.0 ± 2.8
	Friendliness	10.3 ± 3.2	9.6 ± 2.8	9.1 ± 3.3	9.7 ± 3.0	9.6 ± 3.2
	Total Mood Disturbance	26.7 ± 13.8	26.1 ± 17.4	27.7 ± 15.8	27.0 ± 13.4	26.6 ± 17.7

## Data Availability

The datasets generated during and/or analysed during the current study are available from the corresponding author upon reasonable request.
